# Mesenchymal Stromal Cell-Derived Extracellular Vesicles Modulate Hematopoietic Stem and Progenitor Cell Viability and the Expression of Cell Cycle Regulators in an Age-dependent Manner

**DOI:** 10.3389/fbioe.2022.892661

**Published:** 2022-06-01

**Authors:** Pascal Fichtel, Malte von Bonin, Robert Kuhnert, Kristin Möbus, Martin Bornhäuser, Manja Wobus

**Affiliations:** ^1^ Department of Medicine I, University Hospital Carl Gustav Carus, Technische Universität, Dresden, Germany; ^2^ Center for Regenerative Therapies, Technische Universität, Dresden, Germany

**Keywords:** mesenchymal stromal cells, hematopoiesis, bone marrow, extracellular vesicles, aging, hematopoietic stem cells

## Abstract

Aging of the hematopoietic system is characterized by an expansion of hematopoietic stem and progenitor cells (HSPCs) with reduced capacity for engraftment, self-renewal, and lymphoid differentiation, resulting in myeloid-biased hematopoiesis. This process is mediated by both HSPC intrinsic and extrinsic factors, e.g., the stromal environment. A relevant cellular component of the bone marrow (BM) microenvironment are mesenchymal stromal cells (MSCs) which regulate fate and differentiation of HSPCs. The bi-directional communication with HSPCs is mediated either by direct cell-cell contacts or by extracellular vesicles (EVs) which carry bioactive substances such as small RNA, DNA, lipids and proteins. So far, the impact of MSC-derived EVs on human hematopoietic aging is poorly investigated. BM MSCs were isolated from young (n = 3, median age: 22 years) and aged (n = 3, median age: 70 years) donors and the EVs were isolated after culturing the confluent cell layer in serum-free medium for 48 h. CD34^+^ HSPCs were purified from peripheral blood of healthy donors (n = 3, median age: 65 years) by magnetic sorting. Nanoparticle tracking analysis (NTA), transmission electron microscopy (TEM) and western blot detection of EV markers CD63, CD81 and Flotillin-1 revealed no significant differences between young and aged MSC-EVs. Interestingly, young MSCs secreted a significantly higher miRNA concentration than aged cells. However, the amount of distinct miRNAs such as miR-29a and miR-34a was significantly higher in aged MSC-EVs. HSPCs incubated with young EVs showed a significant increase in cell number and a higher viability. The expression of the tumor suppressors PTEN, a known target of mir-29a, and CDKN2A was increased in HSPCs incubated with young EVs. The clonogenic assay demonstrated a decreased colony number of CFU-GM after treatment with young EVs and an increased number of BFU-E/CFU-E after incubation with aged MSC-EVs. Xenogenic transplantation experiments showed no significant differences concerning the engraftment of lymphoid or myeloid cell compartments, but the overall human chimerism 8–16 weeks after transplantation was higher after EV treatment. In conclusion, our data suggest that HSPC characteristics such as cell cycle activity and clonogenicity can be modulated by MSC-derived EVs. Further studies have to elucidate the potential therapeutic relevance of our findings.

## Introduction

Hematopoietic cell transplantation (HCT) often represents the only possible treatment for hematological malignancies including leukemias as well as myelodysplastic syndromes. One major concern is to find a immunogenetically matched donor with age being the second-most relevant determinant of outcome after allogeneic HCT (Kollman et al., 2016; Dehn et al., 2019).

Aging is associated with changes in HSPC function and phenotype, including compromised regenerative potential, loss of quiescence, a deregulated cell cycle and increased metabolic activity, which is accompanied by a significant expansion of the stem cell pool and differentiation skewed towards the myeloid lineage (Akunuru and Geiger, 2016). Consequently, aged HSPCs (donors >50 years) have a reduced capacity to reconstitute hematopoiesis after allogeneic HCT, reducing the pool of HSPC donors (Kollman et al., 2001; Kollman et al., 2016).

Hematopoietic aging is determined by both HSPC intrinsic factors, such as accumulation of DNA damage, replicative stress, and decreased autophagy, as well as extrinsic factors conferred by the endothelial and stromal microenvironment (Ergen, Boles and Goodell, 2012; Kusumbe et al., 2016). However, the mechanisms and cellular components of the niche driving aging remain largely unknown.

A main cellular component of the bone marrow microenvironment (BMME) are mesenchymal stromal cells (MSCs). These multipotent cells can differentiate into adipocytes, chondrocytes and osteoblasts. Importantly, they regulate fate and differentiation of HSPCs and thereby their retention in the BM.

The communication with HSPCs is mediated either by direct cell-cell contacts or by extracellular vesicles (EV). These small membrane-surrounded particles carry different bioactive substances such as small DNA, RNA, lipids and proteins (Baglio et al., 2015; Haraszti et al., 2016). EVs are derived from the endosomal compartment or bud directly from the cell membrane. After incorporation in the recipient cell, they are able to modify the phenotype by transferring proteins or mRNA and changing the gene expression *via* small RNAs (De Luca et al., 2016; Preciado et al., 2019). Therefore, it is suggested that an age-related modification of EV cargo, such as miRNA levels, may have distinct impact on gene expression in the recipient cell.

It was shown that MSC-EV cargo in rats changes with age and that the gene and protein expression of HSPCs is influenced differently after incubation with EVs of young and old murine MSCs (Wang et al., 2015; Kulkarni et al., 2018). Unfortunately, we lack data considering the influence of aging on the EV content of human physiologically aged MSCs and their effects on human HSPCs.

In this study, we characterized EVs of young and aged MSCs and could demonstrate an age-dependent miRNA cargo. We focused on the analysis of miRNAs that are highly abundant in MSC-EVs and play an important role for hematopoiesis (Baglio et al., 2015; Morhayim et al., 2016; Kulkarni et al., 2018). Further, the different ability to support *in vitro* expansion of HSPCs and to influence surface marker expression was evaluated. Important target genes of investigated miRNA regulating differentiation and cell cycling of HSPCs were examined after incubation. Additionally, a potential increased *in vivo* engraftment of HSPCs was analysed by xenogeneic transplantation in NSG mice.

## Materials and Methods

### Isolation and Culture of MSCs

BM MSCs were isolated from young (n = 3, median age: 22 years) and aged (n = 3, median age: 69 years) healthy donors (Supplementary Table S1) after obtaining informed written consent (Ethical Approval No. EK221102004, EK47022007) as described previously (Oswald et al., 2004; Hempel et al., 2016). The cells were expanded in Dulbecco’s modified Eagle’s medium-low glucose (DMEM, Gibco, Germany) with 10% fetal bovine serum (FBS, Gibco) and characterized according to the criteria of the International Society for Cellular Therapy (Dominici et al., 2006). MSCs were used in the second or third passage for all experiments.

### Isolation of CD34^+^ HSPCs

CD34^+^ cells were isolated from leukapheresis of patients (n = 9, 47–67 years old, median age: 52 years) suffering from non-leukemic cancers (e.g., myeloma, lymphoma and sarcoma) after obtaining informed written consent. Stem cells were collected before high-dose chemotherapy in preparation of an autologous HCT. The cells were purified by immunomagnetic sorting using CD34 MicroBead Kit UltraPure (Miltenyi Biotec, Germany). The viability was determined by live-dead-discrimination using Countess II Automated Cell Counter (Thermo Fisher Scientific, Germany) and the purity of at least 98% was confirmed by flow cytometry.

### MSC-EV and MSC-EV-miRNA Isolation

After reaching 80% confluency medium was changed to 100% DMEM. The medium was collected after 48 h and the cells were harvested and counted after staining with trypan blue for live-dead-discrimination. To deplete cell debris the medium was centrifuged at 380xg for 5 min and subsequently filtered through a 200 nm Millipore filter. Afterwards the EVs were isolated with the exoEasy Maxi Kit (Qiagen, Germany) and the EV-miRNA was purified with the exoRNeasy Maxi Kit (Qiagen, Germany) according to the manufacturer’s protocol. Briefly, the sample was mixed with the same volume of buffer XBP and given onto the column. After centrifugation at 500 *g* for 1 min at room temperature the column was washed with buffer XWP and centrifuged at 5.000xg for 10 min. To elute the EVs 400 μl of buffer XE was added onto the column before centrifugation at 500 *g* for 5 min. To increase the number of particles the column was eluted again and centrifuged at 5.000xg for 5 min.

To isolate the miRNA the EVs were bound on the columns analogously, but instead of eluting the particles were lysed in 700 μl Qiazol and the columns were spun down at 5.000xg for 5 min. The total RNA was isolated following the manufacturer’s protocol. The samples were stored at -80°C.

### Nanoparticle Tracking Analysis

The data concerning size distribution and the concentration of the particles were acquired on the ZetaVIEW S/N 243 (Particle Metrix GmbH, Germany). The samples were diluted 1:500 or 1:1,000 in PBS. To process data ZetaView 8.05.05 SP2 was used.

### Western Blot

Eluted EVs were lysed in 10X cell lysis buffer (Cell Signaling Technology, Germany) with protease inhibitor cocktail B (Santa Cruz, United States of America) and were mixed with Roti-Load 2 (Carl Roth, Germany). For reducing conditions DTT was added. 24 μl of the sample was loaded on a 10% or 12% SDS-polyacrylamide electrophoresis gel and transferred to a nitrocellulose membrane. Subsequently, it was blocked in 5% non-fat dry milk and incubated with anti-CD63 (Invitrogen, Germany) and anti-CD81 (Invitrogen, Germany) under non-reducing conditions, anti-Flotillin-1 (BD Biosciences, United States of America) and anti-Calnexin (Cell Signaling Technology, United States of America) under reducing conditions. Afterwards, the membrane was thoroughly washed and incubated with the secondary antibody coupled with a horseradish peroxidase. The chemiluminescence was detected using the ECL Plus Western Blotting Detection Reagent (Amersham, United States of America) on the Luminescent Image Analyzer LAS-3000 (FUJIFILM, Japan).

### Transmission Electron Microscopy

To prepare the samples for Transmission Electron Microscopy (TEM) EV solutions were applied on a 300 mesh EM grid, airdried and fixed with 1% glutaraldehyde. The samples were washed twice with water and counterstained with uranyloxalate solution. Afterwards the EVs were examined under a transmission electron microscope Jeol JEM1400 Plus (Jeol, Germany) running at 80 kV acceleration voltage.

### Quantitative miRNA-RT-PCR

The miRNA amount of the EV samples was calculated with the Qubit™ microRNA Assay Kit (Thermo Fisher Scientific, Germany). cDNA was synthesized using TaqMan^®^ MicroRNA Reverse Transcription Kit (Thermo Fisher Scientific, Germany). RT-PCR was performed using TaqMan^®^ universal PCR Master Mix (Thermo Fisher Scientific, Germany) on a Quantstudio three cycler (Applied Biosystems, Germany). The following miRNAs were examined: let7a, miR-10a, miR-21, miR-23, miR-155, miR-221, miR-222 and miR-486, miR29a, miR34a. All primers were purchased from Applied Biosystems (Supplementary Table S2). The samples were run in duplicates. U18 was used as endogenous control according to Vriens et al. (Vriens et al., 2012). Amplicons were normalized to U18 applying the comparative CT (∆CT) method.

### *In vitro* Incubation of HSPCs With MSC-EVs

2.4 × 10^5^ CD34^+^ HSPCs were incubated with young or aged MSC-EVs collected from 1.2 × 10^5^ cells or XE buffer as control in CellGenix SCGM (CellGenix, Germany) with 10% vesicle-depleted FBS supplemented with SCF, Flt3 ligand and IL-3 (10 ng/ml each, all from Miltenyi Biotec, Germany). The EV samples were pooled beforehand. For vesicle depletion, the FBS was centrifuged with an Amicon-Ultra 100 kDa filter (Millipore, Germany) for 55 min at 3.000xg. Each sample was run in duplicates.

After the incubation, the cells were washed and counted by Countess II Automated Cell Counter (Thermo Fisher Scientific, Germany). Subsequently, samples were pooled and further analyzed using flow cytometry, clonogenic assays and RT-PCR.

### Flow Cytometry

HSPCs cultured with EVs were stained for surface markers using fluorescently labeled antibodies according to Supplementary Table S3. Corresponding human immunoglobulin G controls were used. Data was acquired on a BD FACS Calibur or LSRII. Data were analysed using FlowJo software (FlowJo, LLC, United States of America).

### Fluorescent Labelling and Confocal Microscopy

Isolated EVs were labeled with fluorescent dye 10 µM DiI (Thermo Fisher Scientific). After incubation for 20 min at 37°C the dye was removed with Exosome Spin Columns (Thermo Fisher Scientific). 10^5^ CD34^+^ cells were incubated with DiI-EVs of 3*10^5^ MSCs or buffer as control for 24 h. Afterwards, a cytospin was used to concentrate the cells onto a microscope slide. After fixation in 4% paraformaldehyde, the nuclei were counterstained with DAPI. Images were acquired on a LSM 880 (ZEISS, Germany).

### Clonogenic Assays

Colony‐forming unit (CFU) assays were carried out using CD34^+^ HSPCs harvested after 3 days of incubation with EVs. 500 cells were plated in Stem MACS HSC-CFU complete with Epo (Miltenyi Biotec, Germany). Colonies were counted after 2 weeks and classified with the StemVision system (Stem Cell Technologies, Germany). Each sample was run in duplicates.

### Quantitative RNA-RT-PCR

Total RNA were isolated from CD34^+^ HSPCs after incubation with EVs using RNeasy Micro Kit (Qiagen, Germany) and reverse transcribed into cDNA using RevertAid cDNA synthesis kit (Thermo Fisher Scientific, Germany) with oligo-dT primers. Relative target quantity was determined using the comparative CT (∆∆CT) method. RT-PCR was performed using SYBR Green/ROX PCR master mix (Thermo Fisher Scientific, Germany) and target specific primers for PTEN, CDKN2A and SIRT1 (Supplementary Table S4) on a Quantstudio three cycler (Applied Biosystems, Germany). Amplicons were normalized to endogenous GAPDH control. All samples were run in duplicates.

### Xenogeneic Transplantation

NOD.Cg-Prkdcscid Il2rgtm1Wjl/SzJ (NOD/SCID/IL2rc2/2, NSG) mice were purchased from The Jackson Laboratory (Jackson Laboratory, United States of America). They were kept in the animal facility at the Medical Theoretical Center of the University of Technology Dresden in accordance with German animal welfare legislation after approval of the Landesdirektion Sachsen (TVV 25/2015). Female mice (n = 3 per group) in the age of 8–9 weeks were used for the experiments. CD34^+^ HSPCs were pre-incubated with EVs as described the *in vitro* studies. 1 × 10^5^ cells were injected intravenously after whole body irradiation with 1 Gy. For the first 3 weeks after transplantation, water was supplemented with neomycin (1.17 g/l, Sigma-Aldrich, United States of America). Every 4 weeks peripheral blood was taken and after 16 weeks the animals were sacrificed and bone marrow was collected. All samples were stained with monoclonal antibodies (Supplementary Table S3) after lysis of red blood cells.

### Statistical Analysis

Values were summarized as means, *p* values < 0.05 were considered significant. Statistical analyses including Student’s *t*-Test and One-way ANOVA were calculated with GraphPad Prism 5.00 for Windows.

## Results

### Characterization of MSC-Derived EVs From Young Vs. Aged Donors

After incubating a nearly confluent MSC layer in serum-free DMEM for 48 h, EVs were collected with a column-based isolation method. The MSC viability was proved to be over 95% in all samples. As a reference for EV concentration, the cell number was determined after trypsinization and was 2.4–3.3 × 10^6^ MSCs (median 2.6 × 10^6^).

**FIGURE 1:**
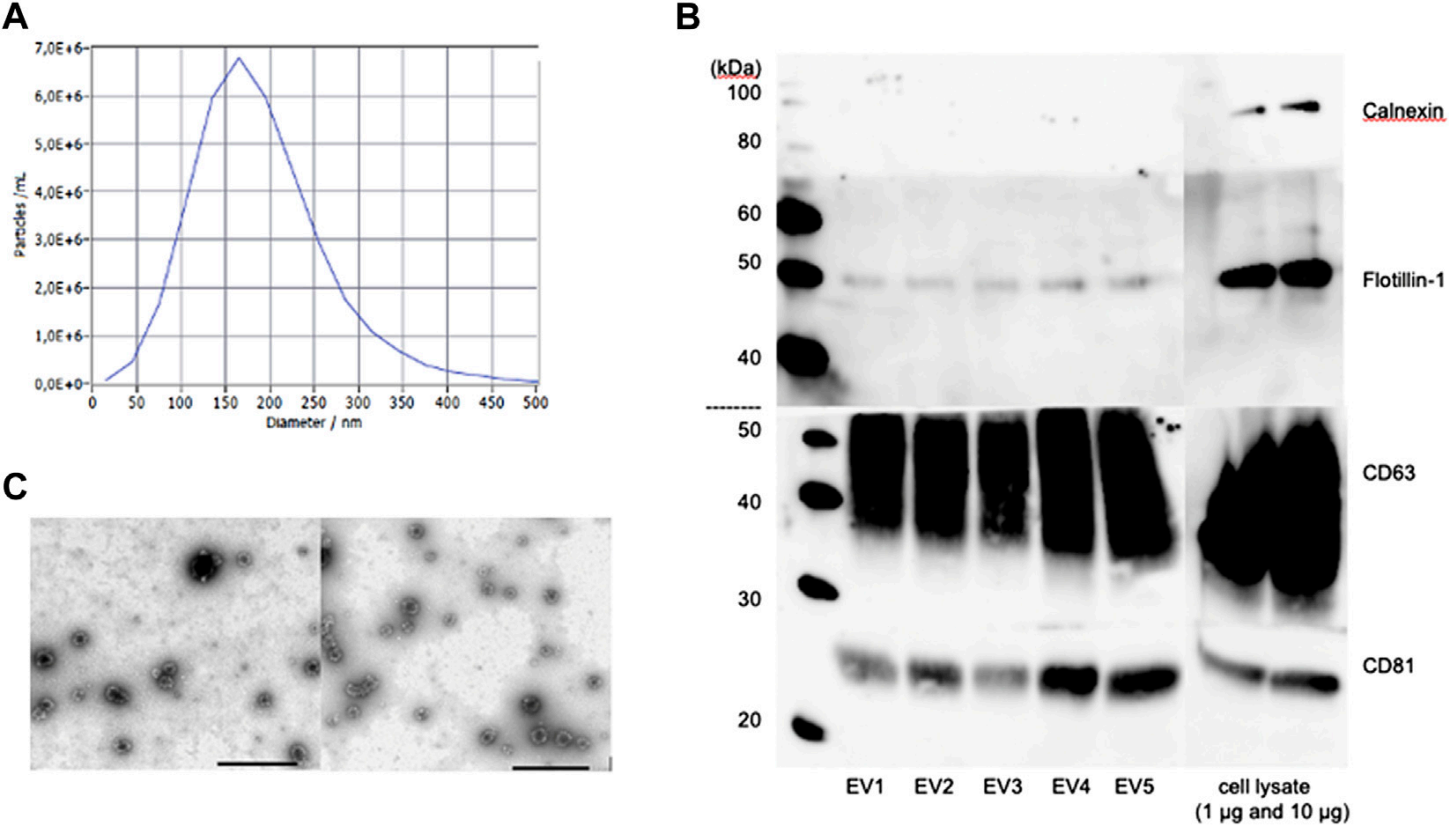
Characterization of MSC-derived EVs of young and aged donors. **(A)** Nanoparticle Tracking Analysis of one representative sample. **(B)** Western Blot analysis of CD63, CD81, Flotillin-1 and Calnexin in different MSC-EV samples. **(C)** Representative TEM images of young MSC-EVs (left) and aged MSC-EVs (right), scale bar: 1,000 nm.

NTA revealed no significant differences in concentration and size distribution between young and aged MSC-EVs. Particles larger than 400 nm could not be detected. The mean of the median diameter of the young and old MSC-EVs (n = 3 per group) was 170.7 ± 8.4 and 174.0 ± 11.0 nm, respectively (Figure 1A). The mean particle concentration was 2,83 ± 0,28 and 2,41 ± 0,49 *10^3^ particles per MSC.

Next, the extracted EVs of young and old MSCs were characterized by Western Blot. The exosomal proteins CD63, CD81 and Flotillin-1 could be detected in all samples, whereas Calnexin, a typical cellular marker, was absent in all samples but present in the cell lysate (Figure 1B). The Transelectron Microscopy showed round vesicular structures with a hypodense center in isolates from both young and aged MSCs. The typical bilayer membrane could be observed. There were no obvious differences in the ultrastructure and the morphology between EVs from young and aged MSCs (Figure 1C).

### Analysis of miRNAs in Young and Aged MSC-EVs

MSCEVs can transfer the cargoes to the recipient cells and thereby alter their activities.

**FIGURE 2:**
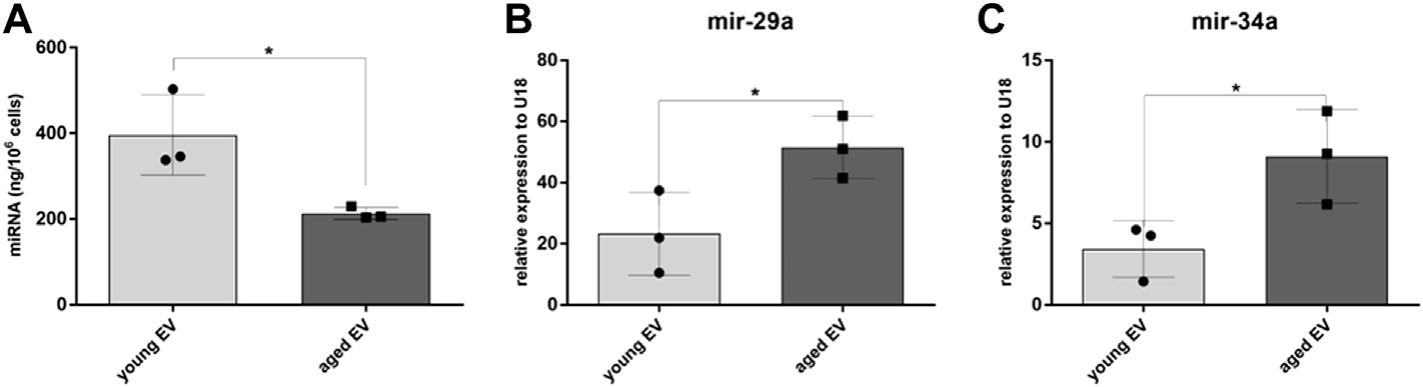
Analysis of miRNA extracted from young and aged MSC-EVs. **(A)** The miRNA amount released per young and aged MSC-EVs was determined by Qubit fluorometry. **(B,C)** Expression of mir-29a and mir-34a in young and aged MSC-EVs was evaluated by quantitative real-time PCR. Data samples were normalized to U18, the bars represent the mean ± SEM from three different MSC donors in each group (n = 3), *p < 0.05.

Since small RNAs are highly abundant in EVs, the miRNA cargo was isolated and the concentration determined by Qubit analysis. Interestingly, the total amount of miRNA was significantly decreased in EVs derived from aged compared to young MSCs (Figure 2A). In a next step a panel of miRNAs relevant for hematopoiesis were quantified by PCR. U18 was used as endogenous control. The Ct value in the group of young MSC-EVs was 29.16 ± 0.96 and in the group of aged MSC-EVs 30.43 ± 0.86 confirming stable U18 expression in both sample cohorts. Whereas no relevant differences could be detected for let-7a, miR-10a, miR-21, miR-23, miR-155, miR-221, miR-222 and miR-486, the levels of miR-29a and miR-34a were significantly higher in EVs from aged MSCs (Figures 2B,C). This suggests an accumulation of specific miRNAs in aged EVs whereas the overall amount was decreased.

### EV Transfer Into HSPCs

**FIGURE 3:**
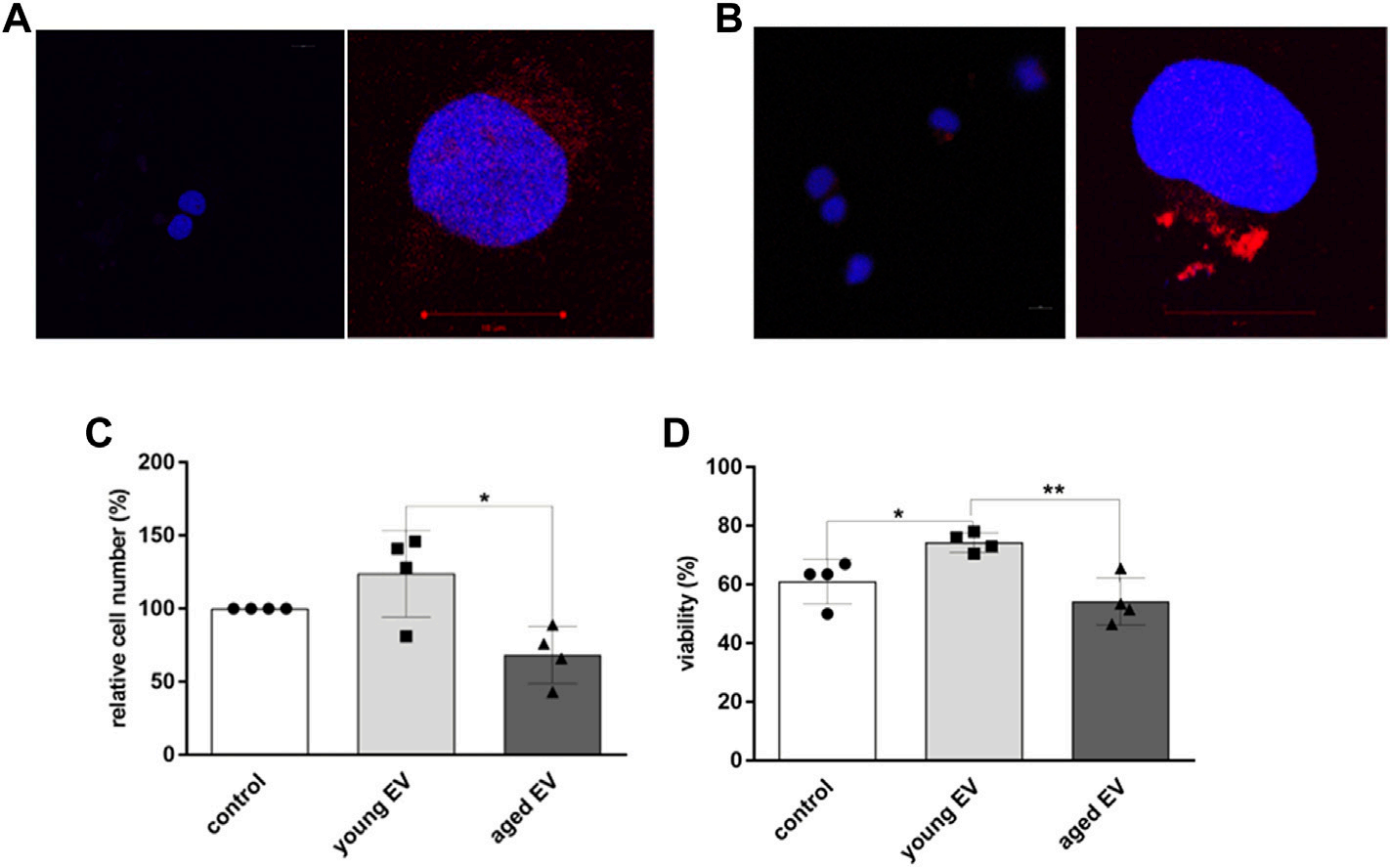
Uptake of MSC-EVs and effects on HSPCs. HSPCs were either treated for 24 h with buffer **(A)** or with Dil-labeled EVs **(B)**. Representative images of confocal light microscopy are shown for lower (×20, left) and higher magnification (×63, right), nucleus of HSPCs is stained with DAPI (blue), EVs are labeled with DiI (red). Scale bar 10 μm. The cell number **(C)** and viability **(D)** was determined by trypane blue staining and counting of HSPCs after 72 h of incubation with young or aged MSC-EVs or buffer as control, respectively. Bars represent the mean ± SEM from four different HSPC donors in each group (n = 3), *p < 0.05, **p < 0.01.

To investigate whether MSC-derived EVs can be incorporated by HSPCs, confocal microscopy was applied. After incubation of HSPCs with Dil labeled EVs for 24 h, an uptake could be detected independent on the age of the parental MSCs. Unstained EVs and buffer, respectively, were used as control (Figures 3A,B).

To analyze the impact of incorporated EVs on HSPC characteristics in more detail, the incubation time was increased for up to 72 h. Aged HSPCs (n = 4) were treated with EVs of young or aged MSCs, respectively, or with XE buffer only as control. EVs derived from young MSCs caused a significant increase in the cell number compared to cells treated with buffer (1.2-fold) or with old EVs (1.8-fold) (Figure 3C). Furthermore, the viability of HSPCs was significantly improved after incubation with young EVs (Figure 3D).

### Gene Expression Analysis in HSPCs After EV Transfer

**FIGURE 4:**
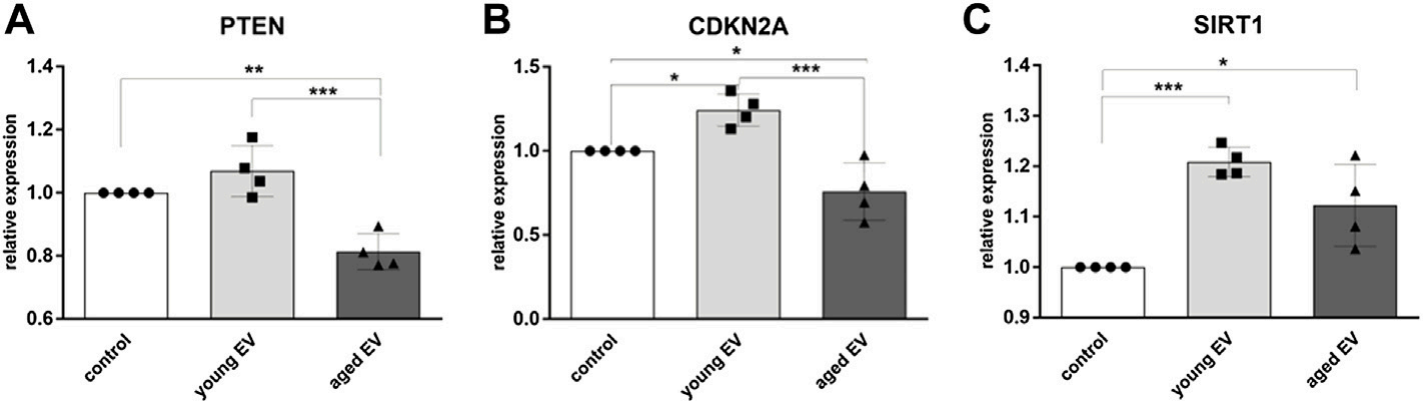
Modulation of gene expression in HSPCs by MSC-EVs. The expression of PTEN **(A)**, CDKN2A **(B)** and SIRT1 **(C)** in HSPCs was evaluated after 72 h of incubation with young or aged MSC-EVs or buffer as control, respectively. Relative target quantity was determined using the comparative CT (∆∆CT) method. Amplicons were normalized to endogenous GAPDH expression and the buffer control was set to 1. Cumulative data from four different HSPC donors are shown as mean ± SEM. Significance was assessed by one-way ANOVA with Tukey’s multiple comparisons test. *p < 0.05, **p < 0.01 ***p < 0.001.

Incorporation of EVs and miRNA release can modulate gene expression of HSPCs. Therefore, the expression of the tumor suppressor gene PTEN, a known target of mir-29a, was decreased in HSPCs treated with aged EVs (Figure 4A). Interestingly, the expression of CDKN2A, encoding for the cell cycle inhibitors p16^INK4A^ and p19, was significantly lower in HSPCs incubated with aged MSC-EVs. In contrast, the gene was upregulated in cells treated with young MSC-EVs (Figure 4B). SIRT1, encoding for the histone deacetylase Sirtuin-1 that regulates a balanced hematopoietic differentiation, is upregulated in both groups receiving EVs (Figure 4C).

Moreover, expression of p53, FOXO3, AKT1, Beclin-1 and ATG12 were investigated. However, no significant differences were detected in the expression levels of these genes.

### Phenotypical Characterization of HSPCs After EV Transfer

**FIGURE 5:**
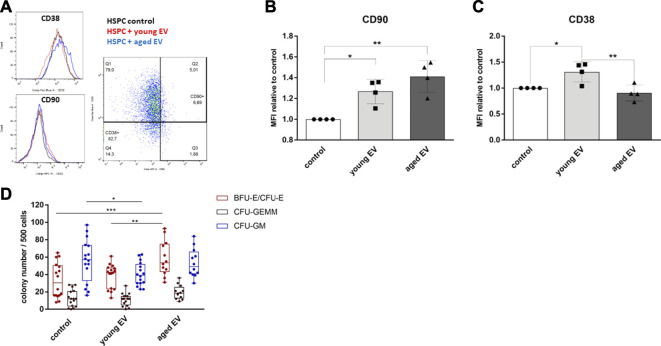
Changes in HSPC surface molecule expression after incubation with MSC-EVs. The expression of CD90 and CD38 in CD34^+^ HSPCs was analyzed by flow cytometry after 72 h of incubation with young or aged MSC-EVs or buffer as control, respectively. **(A)** Representative dot and histogram plots are shown for the detection of CD38 and CD90 expression. **(B)** Graphs show the CD90 and **(C)** CD38 MFI of CD34^+^ cells in relation to their control. **(D)** A CFU-GEMM assay was performed for 14 days in methylcellulose medium and the colonies were classified by using the StemVision system. The bars represent the mean ± SEM from four different HSPC donors in each group, *p < 0.05, **p < 0.01, ***p < 0.001 by two-way ANOVA with Tukey’s multiple comparisons test.

Surface marker expression is an important indicator of differentiation and stemness of HSPCs and contributes to the definition of the functional properties, e.g., after transplantation. Therefore, the expression of the proteins CD38 and CD90 on CD34^+^ population was investigated (Figure 5A). We could not detect significant changes in the absolute expression of these surface markers owing to relevant inter-individual differences. The mean fluorescence intensity (MFI) of the stemness marker CD90 normalized to the respective donor was increased after treatment with MSC-EVs with no respect to the donor’s age (Figure 5B). However, the normalized MFI of CD38, a protein expressed on committed progenitors, was also significantly higher on HSPCs treated with young EVs too (Figure 5C).

### Analysis of the Clonogenic Capacity of EV-Modulated HSPCs

To evaluate the clonogenic potential of HSPCs *in vitro* after EV treatment, colony-forming unit (CFU) assays were performed. The HSPCs of each group were able to differentiate into each type of colony. HSPCs treated with aged MSC-EVs showed a significant higher number of BFU-E/CFU-E colonies. Furthermore, the number of CFU-GM colonies was reduced in the group of HSPCs treated with young MSC-EVs compared to the control group (Figure 5D).

### Evaluation of the *in Vivo* Engraftment Ability of EV-Treated HSPCs

**FIGURE 6:**
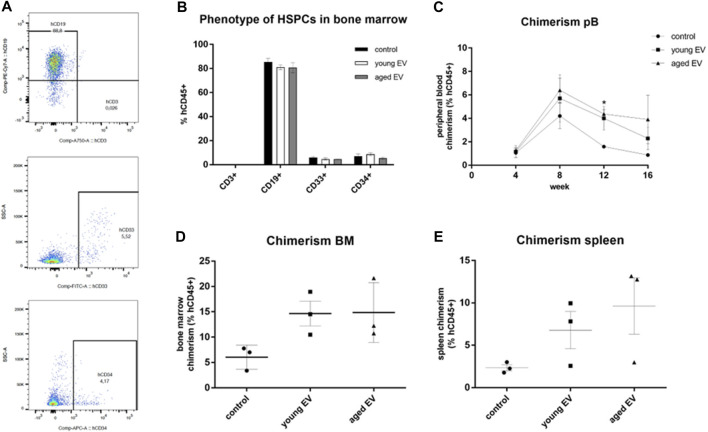
Effects of MSC-EV treatment on HSPC engraftment *in vivo*. HSPCs were treated for 72 h with young or aged MSC-EVs or buffer as a control, respectively, and injected into NSG mice (n = 3 per group). Mice were kept for 16 weeks. Phenotype and chimerism was analyzed by flow cytometry after gating on human CD45^+^ cells. **(A)** Representative dot plots for the detection of human cells expressing CD3, CD19, CD33 or CD34, respectively. **(B)** Phenotype of human CD45^+^ cells in the BM. Lymphoid cells were detected by CD3 (T cells) and CD19 (B cells), myeloid cells by CD33 expression. **(C)** The chimerism defined as percentage of human CD45^+^ cells amongst all CD45^+^ cells was analyzed every 4 weeks in the peripheral blood. **(D,E)** The chimerism in the BM and in the spleen was analyzed 16 weeks after transplantation. The bars represent the mean ± SEM from three different mice in each group, *p < 0.05.

To prove the beneficial effect of MSC-EVs on the engraftment potential of HSPCs *in vivo*, xenotransplantation assays in immunodeficient NSG mice were conducted. 1 × 10^5^ pretreated HSPCs were transplanted. Long-term engraftment could be observed in all three groups assessed by the *de novo* generation of myeloid (CD33^+^) and lymphoid (CD3^+^ and CD19^+^) cells (Figure 6A). There were no significant differences concerning the phenotype and the compartmental attribution of the blood cells neither in the spleen and the peripheral blood nor in the BM (Figure 6B).

The HSPCs treated with aged MSC-EVs showed a significantly higher week 12 peripheral blood chimerism (Figure 6C). The chimerism in bone marrow and spleen was also evaluated after 16 weeks. No significant differences could be observed, although the chimerism was always higher after EV treatment with no respect to the donor’s age (Figures 6D,E).

## Discussion

Aging of HSPCs has a decisive impact on the outcome of HCT, is one of the reasons for decreased immune responses of elderly people and represents an important risk factor for the development of hematologic malignancies (Geiger, De Haan and Carolina Florian, 2013; Kollman et al., 2016). However, aging of HSPCs is not just a cell-intrinsic process, it is also influenced by an aging BMME (Walenda et al., 2010; Kusumbe et al., 2016). To this end, MSCs play a crucial role for the regulation of the HSPC fate. The communication between MSCs and HSPCs is mediated by direct cell-cell-interactions but also by the exchange of EVs. Recent studies have even shown an extension of health span in mice and highlight the meaning of these particles and their role in aging processes (Dorronsoro et al., 2021). However, the age of the MSCs as one factor influencing the potential of their EVs is often neglected in studies and examined just in few studies (Wang et al., 2015; Kulkarni et al., 2018).

We characterized young and aged MSC-EVs according to the recommendations of the International Society for Extracellular Vesicles (Théry et al., 2018). The EV morphology as analyzed by TEM, the protein cargo as examined by Western Blot and the size distribution calculated by NTA showed no difference between the two groups (Figure 1). However, the number of particles produced by each cell is significantly higher in aged MSCs. Fafián-Labora et al. also observed this increase in MSC-EVs of aged rats and hypothesized that it might be a mechanism to increase the disposal of intracellular proteins (Fafián-Labora et al., 2020).

miRNAs represent important mediators in the communication *via* EVs. MSC-EVs show a specific miRNA signature that is altered during aging (Baglio et al., 2015; Fafián-Labora et al., 2017). Interestingly, the total amount of miRNAs decreased in aged MSC-EVs samples what might represent one reason for an impaired ability to support HSPCs. The quantitative analysis revealed a different miRNA profile of young and aged MSC-EVs. The expression of miR-34a, a proapoptotic microRNA regulating the proliferation of cells (Rokavec et al., 2014), was increased in aged MSC-EVs (Figure 2C). Overexpression of miR-34a in HSPCs leads to a myeloid bias and is often detected in patients suffering from myelofibrosis (Bianchi et al., 2017). Moreover, miR-34a is typically upregulated in senescent cells and can be induced by cellular stress (Tazawa et al., 2007; Kulkarni et al., 2018). The expression of miR-29a known as an important smallRNA regulating cell cycle is also increased in aged MSC-EVs (Figure 2B). This miRNA supports the self-renewal of HSPCs, but can also lead to acute myeloid leukemia, when experimentally overexpressed in progenitor cells (Han et al., 2010).

**FIGURE 7:**
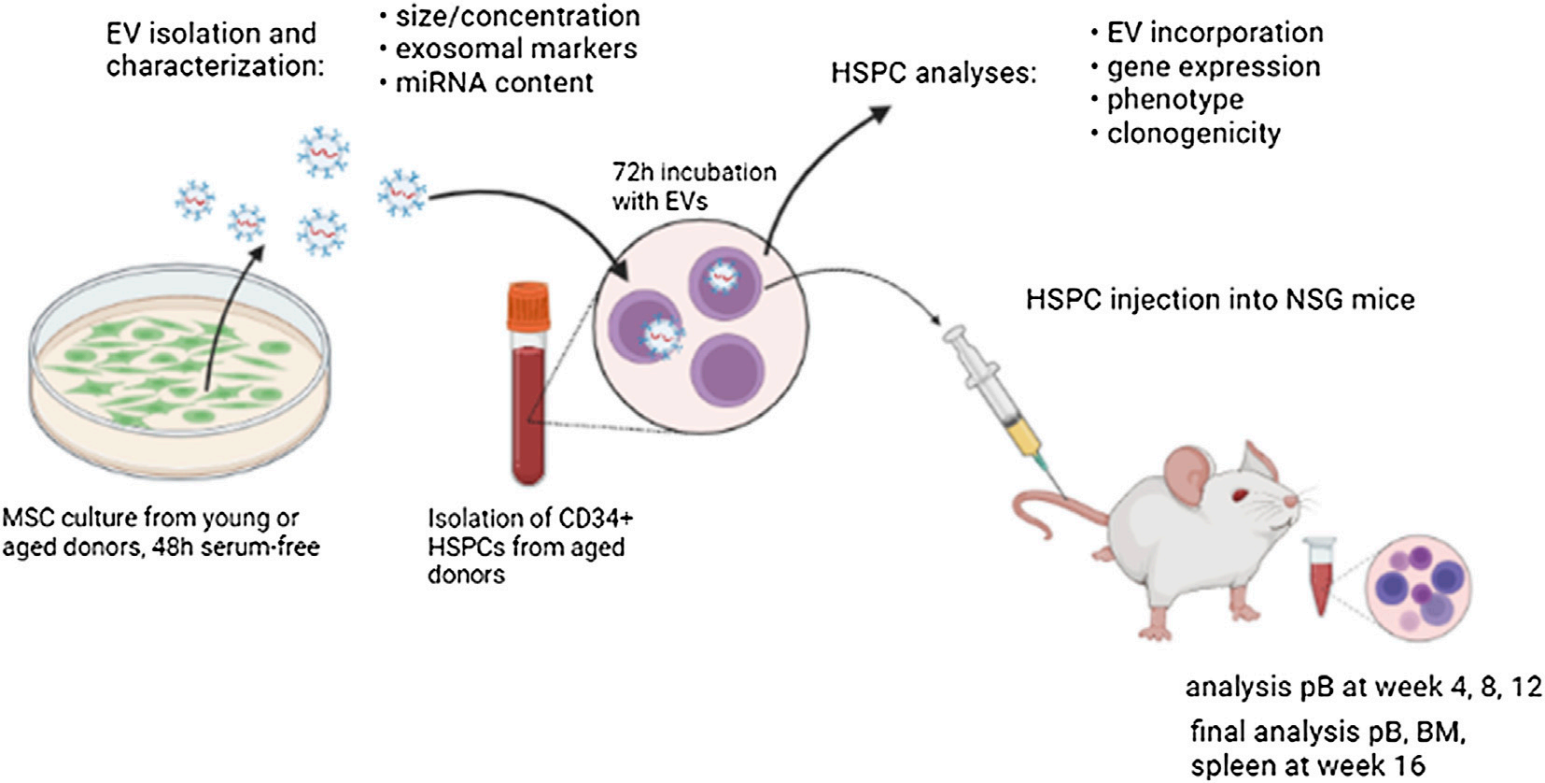
Graphical abstract demonstrating the experimental setup.

In this study, we have examined for the first time effects caused by EVs of physiologically aged human MSCs on HSPCs of elderly people (Figure 7). Interestingly, young MSC-EVs can support the expansion of HSPCs *in vitro*, whereas aged MSC-EVs did not have beneficial effects on the cell survival and the proliferation (Figures 3C,D). This might be the consequence of a well-balanced regulation of cell growth and cell cycling in HSPCs treated with young EVs. Consistent with this assumption, we observed a downregulation of important genes in HSPCs after incubation with aged MSC-EVs. PTEN is a tumor suppressor which regulates cell growth in the AKT signaling pathway and that is important for the reconstitution after HCT (Yilmaz et al., 2006). It is also a target of miR-29a which is upregulated in old MSC-EVs (Morhayim et al., 2016). CDKN2A is another gene that is downregulated in HSPCs after treatment with old MSC-EVs. This gene encodes for proteins that regulate cell cycle especially in aged HSPCs (Furukawa et al., 2000; Janzen et al., 2006). In contrast, SIRT1 is upregulated in HSPCs treated with both young and old MSC-EVs. The encoded protein is a histone deacetylase regulating cell cycle and differentiation of HSPCs (Rimmelé et al., 2014). SIRT1 is a component in young and aged murine MSC-EVs (Kulkarni et al., 2018). Thus, this mRNA is likely to be found in human MSC-EVs and may be transferred directly *via* EVs.

Consistent with this finding the MFI of CD90 increased after the treatment with MSC-EVs regardless of donor age. CD90 represents a marker indicating the stemness of HSPCs. However, it can also be found on MSCs and MSC-EVs (Ramos et al., 2016). An increased CD90 expression has been observed after culturing HSPCs on a MSC layer (Notta et al., 2011) which supports the hypothesis of a direct transfer of this protein *via* EVs.

The clonogenic assays revealed a restrictive effect of young MSC-EVs on the myeloid differentiation (Figure 5D). This supports the idea that especially the young BMME regulates the stem cell fate and avoid a myeloid bias observed in aged individuals (Dykstra et al., 2011; De Luca et al., 2016). Surprisingly, aged MSC-EVs seem to support the differentiation of HSPCs into erythroid colonies.

It is known that the engraftment of HSPCs is improved after co-culturing on a MSC layer (Huang et al., 2007; Bernardo, Cometa and Locatelli, 2012). However, the mechanism of support is not fully understood. Our results suggest EVs as one potential mediator to improve the engraftment indicated by a higher chimerism especially in the peripheral blood. In contrast to findings with murine cells, the effect does not seem to depend on the MSC donor’s age (Kulkarni et al., 2018).

## Conclusion

In conclusion, we provide evidence that human MSC-derived EVs underlie age-related changes in terms of their molecular cargo which might negatively impact recipient cells. Consequently, only young MSC-EVs supported the proliferation and viability of HSPCs *in vitro* and enhanced the expression of genes regulating cell growth and division. Additionally, MSC-EVs improved the engraftment of HSPCs *in vivo*. These data indicate a potential therapeutic relevance of MSC-EVs and may stimulate further translational studies to evaluate their role in age-related pathologies.

## Data Availability

The original contributions presented in the study are included in the article/Supplementary Material, further inquiries can be directed to the corresponding authors.
