# Supplementary material for: Mesenchymal Stromal Cell-Derived Extracellular Vesicles Modulate Hematopoietic Stem and Progenitor Cell Viability and the Expression of Cell Cycle Regulators in an Age-dependent Manner
Source: Front Bioeng Biotechnol. 2022 Jun 1;10:892661. doi: 10.3389/fbioe.2022.892661 (PMC9198480; doi:10.3389/fbioe.2022.892661)
Supplement: Supplementary file 1 [file DataSheet1.docx]

**Supplementary Material**

**Table 1. MSC donor information**

|  | number | sex | age |
| --- | --- | --- | --- |
| young donor | #1 | female | 23 |
|  | #2 | female | 22 |
|  | #3 | female | 21 |
| aged donor | #4 | female | 69 |
|  | #5 | female | 63 |
|  | #6 | female | 79 |

**Table 2. Primer sequences miRNA**

| **miRBase ID** | **miRBase accession number** | **sequence** |
| --- | --- | --- |
| hsa-let-7a-5p | MIMAT0000062 | UGAGGUAGUAGGUUGUAUAGUU |
| hsa-miR-10a-5p | MIMAT0000235 | UACCCUGUAGAUCCGAAUUUGUG |
| hsa-miR-21-5p | MIMAT0000076 | UAGCUUAUCAGACUGAUGUUGA |
| hsa-miR-23a-3p | MIMAT0000078 | AUCACAUUGCCAGGGAUUUCC |
| hsa-miR-29a-3p | MIMAT0023884 | UAGCACCAUCUGAAAUCGGUU |
| hsa-miR-34a-5p | MIMAT0038095 | UGGCAGUGUCUUAGCUGGUUGUU |
| hsa-miR-155-3p | MIMAT0004658 | CUCCUACAUAUUAGCAUUAACA |
| hsa-miR-221-3p | MIMAT0000278 | AGCUACAUUGUCUGCUGGGUUUC |
| hsa-miR-222-3p | MIMAT0000279 | AGCUACAUCUGGCUACUGGGU |
| hsa-miR-486-3p | MIMAT0004762 | CGGGGCAGCUCAGUACAGGAU |

**Table 3. Flow cytometry antibodies**

| antigen | fluorophor | species | company | purpose |
| --- | --- | --- | --- | --- |
| CD34 | FITC | human | Miltenyi Biotec | HSPC isolation |
| CD38 | PE | human | R&D | HSPC isolation |
| CD90 | APC | human | eBioscience | HSPC isolation, in vitro EV incubation |
| CD34 | PerCP-Cy5.5 | human | BD Bioscience | in vitro EV incubation |
| CD38 | Bv421 | human | BD Bioscience | in vitro EV incubation |
| CD45 | PE | mouse | eBioscience | xenogeneic transplantation |
| CD45 | V500 | human | BD Bioscience | xenogeneic transplantation |
| CD3 | APC-eFluor780 | human | eBioscience | xenogeneic transplantation |
| CD19 | PE-Cy7 | human | BD Bioscience | xenogeneic transplantation |
| CD33 | FITC | human | eBioscience | xenogeneic transplantation |
| CD34 | APC | human | BD Pharming | xenogeneic transplantation |

**Table 4. Primer sequences**

| gen | sequence 5’-3’ | |
| --- | --- | --- |
| GAPDH | forward | GAAGGTGAAGGTCGGAGTC |
|  | reverse | GAAGATGGTGATGGGATTTC |
| SIRT1 | forward | CCAGATCCTCAAGCGATGTT |
|  | reverse | AGAGATGGCTGGAATTGTCC |
| CDKN2A | forward | CAAGGTCCCTCAGACATCCCC |
|  | reverse | CCCTGTAGGACCTTCGGTGAC |
| PTEN | forward | CCACAGCTAGAACTTATCAAACCCT |
|  | reverse | TCATTACACCAGTTCGTCCCTTTC |
